# *Pachygenium laurense* (Orchidaceae, Spiranthinae), a new orchid species from Argentina—morphological evidence and phylogenetic reconstruction

**DOI:** 10.7717/peerj.13433

**Published:** 2022-05-26

**Authors:** Claudia M. Martin, Adriana Marisel Morales, Magdalena Dudek, Dariusz L. Szlachetko

**Affiliations:** 1 Facultad de Ciencias Agrarias, Universidad Nacional de Jujuy, Jujuy, Argentina; 2 Instituto de Ecorregiones Andinas (CONICET-UNJu), Universidad Nacional de Jujuy, Jujuy, Argentina; 3 Department of Plant Taxonomy and Nature Conservation, University of Gdansk, Gdansk, Poland

**Keywords:** Argentina, Field study, Morphology, New species, Orchidaceae, Phylogenetic analyses, *Pachygenium*

## Abstract

**Background:**

*Pachygenium* embraces a group of terrestrial species formerly placed in *Pelexia* sensu lato. The genus currently comprises some 60 species, most of which are known from the southern parts of Brazil and Paraguay, with few species distributed in the Andean countries—only four species have been recorded from Argentina so far. In Jujuy Province, Argentina a new species of *Pachygenium* was found during our fieldwork. The aim of this article was to provide morphological and molecular evidence for its membership in this genus.

**Methods:**

Materials from specimens were collected in the field and examined by classical taxonomic and molecular biological techniques, *e.g.*, PCR and sequencing DNA. Phylogenetic reconstruction was performed by maximum-likelihood and Bayesian inference.

**Results:**

*Pachygenium laurense* from Argentina is described and illustrated based on morphological evidence and its taxonomic position was confirmed by phylogenetic analyses. A new combination for *Pachygenium gutturosa* is also proposed. A key for identification is provided for the *Pachygenium* species occurring in Argentina.

**Conclusion:**

*Pachygenium laurense* is the fifth species of the genus recorded from Argentina.

## Introduction

The name *Pachygenium* was established by Schlechter (1920), who distinguished it as one of the five sections of the genus *Pelexia* Poit. *ex* Lindley (Orchidaceae, Spiranthinae). Based mainly on the spur structure, Schlechter recognized, aside from *Pachygenium*, the following sections: *Cogniauxiocharis*, *P otosia*, *Eupelexia*, and *Centropelexia*. The genus *Pelexia*, in its broad circumscription, is difficult to define in the terms of morphology. As a result, the taxonomic status of some of the aforementioned sections has changed. *Cogniauxiocharis* was incorporated in *Pteroglossa* Schltr. (Garay, 1982), and *Potosia* was erected to the generic level by Tamayo & Szlachetko (Mytnik, 2003); Szlachetko, González & Rutkowski (2001) proposed *Pachygenium* to be assigned the rank of genus, which was subsequently supported by molecular analyses (Salazar et al., 2018).

*Pachygenium* embraces a group of terrestrial species which are rather easily distinguished from *Pelexia* s.s. by several characters. In all species of this genus the leaves have narrow, cuneate blades gradually transforming into indistinct petioles (*vs.* blade ovate to cordate with cordate to truncate base, distinctly diverse from the very narrow petiole). The spur is saccate and short, usually reaching the middle of the ovary (*vs.* spur usually cylindrical, acute at apex, often equal in length to or even longer than pedicel and ovary). The lateral sepals of *Pachygenium* are deeply saccate at the base, a feature lacking in *Pelexia* s.s. The strongly S-curved basal part of the lip of *Pachygenium* effects that the auricles are perpendicular to the ovary axis. Unlike this, the auricles and basal part of the lip of *Pelexia* s.s. are parallel to the ovary. A short and massive gynostemium with short and relatively wide rostellum are characteristics of *Pachygenium*, and different in *Pelexia* s.s., where the gynostemium is elongate, slender, with a narrow and linear rostellum. The viscidium in the former is transversely elliptic and solid, and in the latter small and delicate.

As thus defined, *Pachygenium* currently counts about 60 species (Mytnik-Ejsmont, Szlachetko & Górniak, 2010). The greatest diversity of the genus occurs in the southeastern and southern regions of Brazil, with a secondary center of diversity in Paraguay and only few species reported from Uruguay and the Andean countries.

According to the Flora of Argentina (http://www.floraargentina.edu.ar) the genus *Pelexia* s.l. is represented in that country by eight species, of which four are classified as belonging to *Pachygenium*: *P. bonariense* (Lindl.) Szlach., R. González & Rutk., *P. ekmanii* (Kraenzl.) Szlach., R. González & Rutk., *P. ovatifolium* (M.N. Correa) Szlach., R. González & Rutk., and *P. paludosum* (M.N. Correa) Szlach., R. González & Rutk. The fifth species, *P. saltense* (Griseb.) Szlach., R. González & Rutk., is often treated as a synonym of *P. bonariense* (Szlachetko, González & Rutkowski, 2001).

Our field studies on the biodiversity of plants in Argentina resulted in the discovery of a new species of orchid. The analyses based on morphological features indicate its membership in the genus *Pachygenium*. Moreover, phylogenetic reconstruction was performed using nuclear and plastid markers that also support this result. Its morphology does not match the characters of any other known representative of the genus. The aim of this study was to describe this species as new and to provide key for identification to all species of *Pachygenium* from Argentina.

## Materials & Methods

### Morphological study

All materials collected in the field were examined according to standard procedures. Each studied sample was photographed, and the flowers were placed in liquid. The form, size, and surface of leaves, tubular sheaths enveloping the scape, as well as details of the inflorescences (including form of the floral bracts and ovaries) were examined. The perianth segments were scrutinized and measured under a stereoscopic microscope. The obtained data were compared with original diagnoses and illustrations of representatives of *Pelexia* and *Pachygenium* from Argentina and adjacent countries. We examined over 1000 specimens of these genera stored in AAU, AMES, AMO, B, BA, BIGU, BM, C, COAH, COL, E, F, G, GOET, K, L, M, MO, P, S, SEL, SP, U, US, W, WRSL, WU, and Z.

Information on the occurrence and habitats of the new species was verified during field studies conducted in Argentina in 2020 and 2021. All field experiments complied with provincial, state, and national laws. The Dirección Provincial de Biodiversidad de Jujuy–Secretaría de Gestión Ambiental (Jujuy, Argentina) provided permission to conduct the research under permit number 171/2015-DPB.

“The electronic version of this article in Portable Document Format (PDF) will represent a published work according to the International Code of Nomenclature for algae, fungi, and plants (ICN), and hence the new names contained in the electronic version are effectively published under that Code from the electronic edition alone. In addition, new names contained in this work which have been issued with identifiers by IPNI will eventually be made available to the Global Names Index. The IPNI LSIDs can be resolved and the associated information viewed through any standard web browser by appending the LSID contained in this publication to the prefix “http://ipni.org/”. The online version of this work is archived and available from the following digital repositories: PeerJ, PubMed Central SCIE, and CLOCKSS”.

### Molecular analyses

For phylogenetic reconstruction we applied 84 sequences of the ITS region, 90 of the trnL-trnF marker, and 75 of the matK gene representing taxa from seven genera: *Cyclopogon*, *Veyretia*, *Glohisarcon*, *Sarcoglottis*, *Pelexia*, *Brachystele*, and *Pachygenium*. In selecting the samples for ours analyses, we relied on the results obtained by Salazar et al. (2018). To determine the phylogenetic relations for the new taxon, we used species that grouped together with *Pachygenium* and *Pelexia*. Salazar et al. (2018) showed that representatives of *Cyclopogon* and *Veyretia* appear as a sister group, therefore we also used these as samples. *Cyclopogon obliquus* was selected to root the trees.

Most of the sequences used in this article were downloaded from GenBank (http://www.ncbi.nlm.nih.gov/). A list of the taxa with their accession numbers is included in Appendix 1. Samples (leaf fragments) of *Pachygenium laurense* used for molecular studies were collected in the field by CM. The exact localities with numbers for names and collector are presented in Table 1. GenBank accession numbers for the sequences for these samples are listed in Table 2.

**Table 1:** Locality of samples of *Pachygenium laurense* which were used on molecular analyses.

Taxon	Locality	Date	Collector	No
*Pachygenium laurense* 1	ARGENTINA. Jujuy, Dept. San Pedro. San Juan de Dios, Finca Las Lauras [S S24°33′49.43″; W64°39′17.77″; 941msl]	20 Feb 2021	Claudia M. Martín	2873
*Pachygenium laurense* 2	ARGENTINA. Jujuy, Dept. San Pedro. San Juan de Dios, Finca Las Lauras [S 24°33′48.13″; W64°39′23.63″; 916msl]	20 Feb 2021	Claudia M. Martín	2874
*Pachygenium laurense* 3A	ARGENTINA. Jujuy, Dept. San Pedro. San Juan de Dios, Finca Las Lauras [S 24°33′39″; W 64°39′26,9″; 906msl]	21 Feb 2021	Claudia M. Martín	2875
*Pachygenium laurense* 3B	ARGENTINA. Jujuy, Dept. San Pedro. San Juan de Dios, Finca Las Lauras [S 24°33′39″; W 64°39′26,9″; 906msl]	21 Feb 2021	Claudia M. Martín	2876

**Table 2:** The accession numbers of GenBank for samples of *Pachygenium laurense*.

Taxon	No GenBank of ITS	No GenBank of matK gene	No GenBank of trnL-trnF
*Pachygenium laurense* 1	OL619328	OL694863	OL631601
*Pachygenium laurense* 2	OL619327	OL694861	OL631600
*Pachygenium laurense* 3A	OL619330	—–	OL631599
*Pachygenium laurense* 3B	OL619329	OL694862	OL631602

**DNA Isolation.** Total genomic DNA was extracted from 20–100 mg of dried leaves (Chase & Hills, 1991) using the DNA Sherlock AX Kit (A&A Biotechnology, Poland) following the manufacture’s protocol. The pellets of DNA were suspended in 50 µl of TE buffer.

**Amplification and sequencing.** The PCRs and sequencing reactions were performed for three markers, two plastid (*mat*K and *trn*L-*trn*F) and one nuclear (ITS1+5.8S+ITS2). In both, the same pairs of primers for each marker were used: for the ITS region, 101F and 102R primers (Douzery et al., 1999) while for the plastid region *trn*L-F containing the *trn*L intron and *trn*L-*trn*F intergenic spacer using primers *trn*L-c and *trn*L-f as described by Taberlet et al. (1991), and for the part of the *mat*K gene, primers 19F (Molvray, Kores & Chase, 2000) and 1326R (Cuénoud et al., 2002).

The total volume of sample for amplification was 25 µl containing 1 µl template DNA (∼10–100 ng), 0.5 µl of 10 µM of each primer, 11 µl MyTaq HS DNA Polymerase Mix (BIOLINE Ltd., UK), and water. Parameters for the PCR reaction for nrITS (ITS1+5.8S+ITS2) were: 94 °C, 4 min; 30 × (94 °C, 45 s; 52 °C, 45 s; 72 °C, 1 min), and 72 °C, 7 min. For plastid markers, conditions for the *mat*K gene were: 95 °C, 3 min; 33 × (94 °C, 45 s; 52 °C, 45 s, 72 °C, 90 s); 72 °C, 7 min, and for the *trn*L-F region: 94 °C, 3 min; 35 × (94 °C, 60 s; 55 °C, 60 s, 72 °C, 120 s); 72 °C, 7 min. All products of the PCR reaction were tested using electrophoresis in 1% agarose gel with at 110 V for 25 min. The Clean-Up Concentrator Kit (A&A Biotechnology, Poland) was used to clean the PCR products following the manufacturer’s protocol, and eluted with 30 µl of nuclease-free water.

Purified PCR products were sequenced by Macrogen (Seoul, South Korea - http://dna.macrogen.com/eng/), using the same primers mentioned above. DNA sequence chromatograms were examined/edited in FinchTV (https://finchtv.software.informer.com/1.4/).

**Phylogenetic reconstruction.** First, multiple sequence files for single markers were aligned with SeaView (Galtier, Gouy & Gautier, 1996). We used “align” option according the MUSCLE algorithm (Edgar, 2004). All alignments were inspected, possibly corrected and trimmed to equal length with SeaView (Galtier, Gouy & Gautier, 1996). Maximum-likelihood (ML) and Bayesian inference (BI) were used for phylogenetic reconstruction to test for possible topological incongruence. Best fit substitution models were obtained with the jModelTest 2 (Darriba et al., 2012) (GTR+G+I for the *mat*K matrix and GTR+G for the datasets ITS and *trn*L-*trn*F).

The ML/Thorough bootstrap workflow analysis was performed using RAxML-HPC2 (Stamatakis, 2014) by searching for the best-scoring ML tree under the previously calculated nucleotide substitution model. The branch support values (BS) were calculated with RaxML by halting bootstrapping automatically under the autoMRE criterion.

Bayesian inference was performed using MrBayes *v.* 3.2.7a (Ronquist et al., 2012). Two independent runs of four Markov-chain Monte Carlo (MCMC) chains (one heated and three cold) were started from different random trees to ensure that individual runs had converged to the same result. We used 2 million generations for ITS and *mat*K matrices, and 3 million for the *trn*L-*trn*F dataset per run with sampling every 100 generations. Convergence was assessed using the average standard deviation of split frequencies below 0.01 and the effective sample size (ESS) was checked in the Tracer *v.* 1.7.1 (Rambaut et al., 2018). Thereafter we discarded the initial 25% of the sampled generations of each chain as burn-in. Saved trees were summarized in a majority rule consensus tree and were edited with FigTree v.1.4.4 (http://tree.bio.ed.ac.uk/software/figtree/) and Inkscape (https://inkscape.org/release/inkscape-1.0.2/). The nodal confidence was assessed by posterior probabilities (PP), which were considered strongly supported when equal to or higher than 0.95 (Huelsenbeck & Ronquist, 2001).

## Results

**Figure 1:**
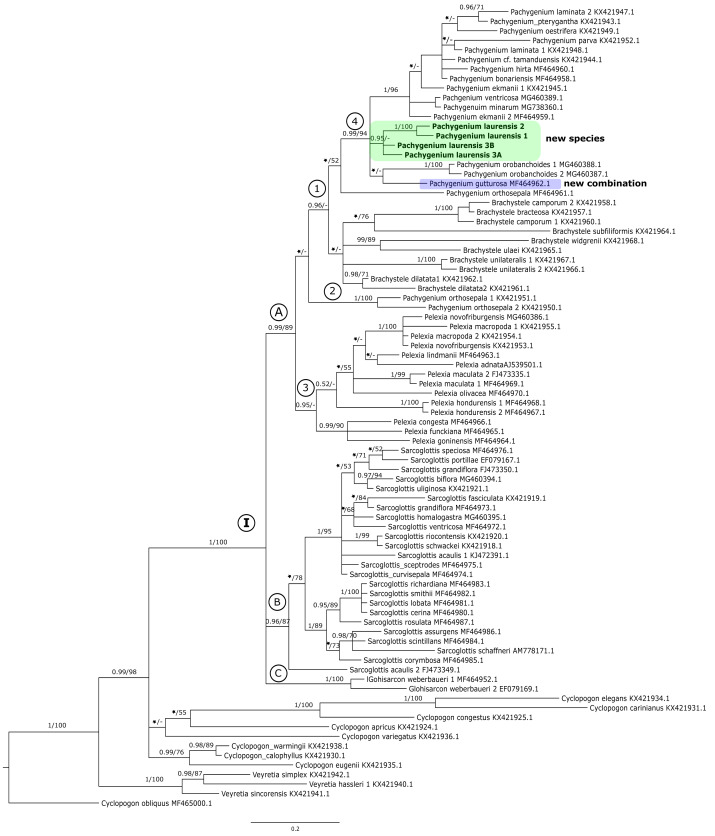
The 50% majority-rule consensus tree obtained for the ITS marker showing the position of *Pachygenium laurense*. Posterior probability (PP) and bootstrap support (BS) values are indicated above the branches; BS values of <50% as –, PP < 0.95 as an asterisk.

**Figure 2:**
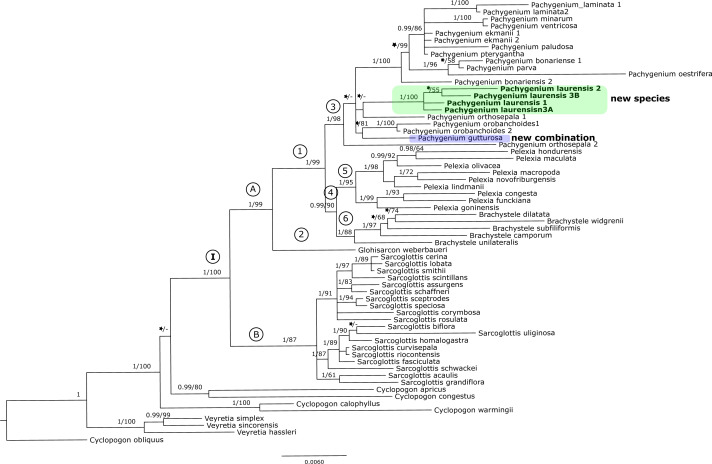
The 50% majority-rule consensus tree obtained for the combined matrix of plastid regions showing the position of *Pachygenium laurense*. Posterior probability (PP) and bootstrap support (BS) values are indicated above the branches; BS values of <50% as –, PP < 0.95 as an asterisk.

**Figure 3:**
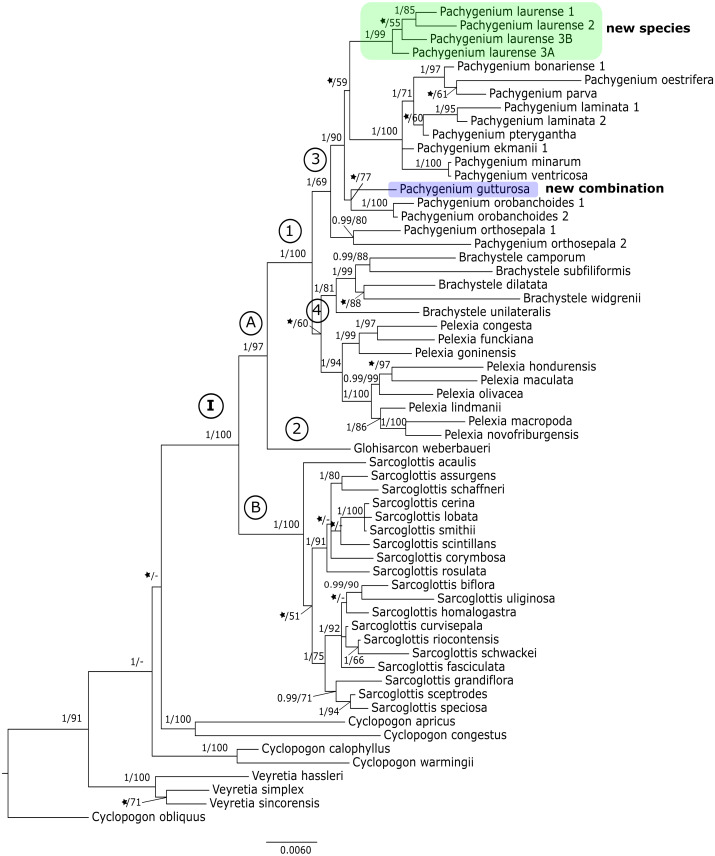
The 50% majority-rule consensus tree obtained for the combined matrix of nuclear and plastid regions showing the position of *Pachygenium laurense*. Posterior probability (PP) and bootstrap support (BS) values are indicated above the branches; BS values of < 50% as –, PP < 0.95 as an asterisk.

The results of the maximum-likelihood and Bayesian inference gave similar results. Thus, we present and discuss the 50% majority-rule consensus tree only. The clades that have low support for bootstrap values in the trees of the ML analyses also have low posterior probability values in trees resulting from Bayesian inference. The topology of trees obtained for the *mat*K and the *trn*L-*trn*F regions are similar. Therefore, we decided to perform combined analyses for these markers. We also made a combined analysis for all used fragments of DNA. In this article we present the trees from the ITS region (Fig. 1) and combined matrices (Figs. 2–3). All other obtained trees are available from the corresponding author. Table 3 lists the statistical data.

### ITS matrix

In the ITS tree representatives of *Cyclopogon* and *Veyretia* were placed at its base (Fig. 1). One of species of *Cyclopogon* (*C*. *obliquus*) served as an outgroup. The genus *Veyretia* is represented by three species in our analyses and all of them are included in one strongly supported group at the base of the tree. The examined species of *Cyclopogon* did not form a coherent clade and were recovered in a polytomy with clade **I** (Fig. 1). The main clade **I** of this tree embraces representatives of *Glottisarcon*, *Sarcoglottis*, *Pelexia*, *Brachystele*, and *Pachygenium*. All these taxa appear to be closely related as evidenced by strong support (PP = 1, BS = 100). In clade **I** we can recognize three smaller groups (clades **A**-**C**, Fig. 1). The first one (clade **A**) with strong support, both posterior probability and bootstrap value (PP = 0.99, BS = 89) includes species of *Pelexia*, *Brachystele*, and *Pachygenium*. Representatives of *Pelexia* (subclade **3**, Fig. 1) formed a consistent group with strong support but only by posterior probability (PP = 0.95). The results of the ML analysis did not yield any support for this clade. We observed a similar situation in subclade **1** that embraces taxa of *Pachygenium* and *Brachystele*. It is worth noting that samples of *Pachygenium orthosepala* are not gathered in one clade. Two of them (KX421950, KX421951) were placed together (Fig. 1, subclade **2**). The third sample of *P*. *orthosepala* (MF464961) is included in subclade 1 with other *Pachygenium* and *Brachystele* species. The results of the BI analysis showed that species of *Brachystele* and *Pachygenium*, expect *P*. *orthosepala* 1 and 2, are grouped together with strong support (subclade **1**, PP = 0.96, Fig. 1). However, this is not supported by the results of the ML analysis, and it is the only difference in the results in these methods. *Pachygenium laurense* in our study was represented by four samples which form a strongly supported group (PP = 0.95) and it is placed with other representatives of *Pachygenium* (subclade **4**, Fig. 1).

Clade **B** embraces taxa of *Sarcoglottis*, while the samples of *Glottisarcon weberbaueri* formed clade **C** (Fig. 1). Both these groups are strongly supported (PP = 0.96, BS = 87; PP = 1, BS = 100) and closely related to representatives of clade **A**.

### Combined matrices (*mat*K *+ trn*L*-trn*F and ITS + plastid markers)

The trees obtained for the combined matrices showed similar results as for the nuclear marker. Again, *Cyclopogon obliquus* is an outgroup, whereas species of *Veyretia* and the other sampled species of *Cyclopogon* were placed at the base of this phylogram. The main group of this tree (clade **I**, Figs. 2–3) embraces representatives of *Glottisarcon*, *Sarcoglottis*, *Pelexia*, *Brachystele*, and *Pachygenium*. Clade **I** was strongly supported both by posterior probability and bootstrap value (PP = 1, BS = 100) and it is divided into two smaller groups. The first, clade **A,** (Figs. 2–3) indicated a sister relationship between the taxa of *Pelexia*, *Brachystele*, and *Pachygenium* (clade **1**) and *Glottisarcon weberbueri*. The members of *Pachygenium*, including the new species, formed the monophyletic clade **3** (Figs. 2–3) with strong support, both by posterior probability and bootstrap values. *P*. *laurense* is represented by four samples, all of which grouping together with one clade of high bootstrap support (BS = 100) and posterior probability (PP = 1).

**Table 3:** Statistical data for all matrices used in the phylogenetic analyses.

Matric/Data	No of taxa	Total characters	Constant Characters	Informative characters
ITS1-5.8S-ITS2	84	823	542	163
part of *mat*K gene	75	1881	1399	186
*trn*L-*trn*F region	90	1440	1052	182
combined of plastid markers	63	3247	2567	272
combined of all markers	60	4034	3132	392

The species of *Pelexia* and *Brachystele* (clade **4**) (Figs. 2–3) run as two consistent clades, sisters to each other, and both are strongly supported (PP = 1, BS = 98; PP = 0.99, BS = 88). The second one, clade **B,** includes species of *Sarcoglottis* (Figs. 2–3).

## Discussion

The new species described in this article is an example of an unusual combination of various characters. While in flowering, *Pachygenium laurense* brings to mind *Porphyrostachys parviflora* (C. Schweinf.) Garay from Peru, especially with its leafless stem and densely many-flowered inflorescences. The ecallose, shortly clawed lip resembles that of *Eltroplectris* Raf., but the relatively slender elongate gynostemium appears to be more similar to that of *Pelexia* s.s. The narrow leaf blade gradually transiting into the petiole, and the saccate spur suggests a relationship with *Pachygenium*.

Our analyses of molecular markers have been able to help to clarify the taxonomic position of this odd species. The phylogenetic trees obtained for the single markers (*i.e.,* ITS, *mat*K, and *trn*L-*trn*F) (Fig. 1) and the combined matrices (Figs. 2–3) reveal that this taxon is accommodated in the *Pachygenium* clade. There are several characters, however, that are unique for this species and unknown for other species of *Pachygenium* as described to date. There are some species of *Pachygenium* growing in drier areas which can have either well-developed leaves or leaves withering in flowering. At the best of our knowledge, *P. laurense* appears to be the only species of the genus in South America which is totally leafless at anthesis. Interestingly, *Pelexia gutturosa* Rchb.f. from Mesoamerica, has very similar leafless habit and general flower architecture. Furthermore, *P. gutturosa* also grouped with the *Pachygenium* clade in our analysis (clade **4**, Fig. 1 and clade **3** Figs. 2–3). Therefore, we decided to transfer this species to the genus *Pachygenium* as well.

The other unique features of the new species are: very characteristic arrangements of the flowers in the inflorescence, ecallose lip, undivided into epichile and hypochile and narrow gynostemium. In our opinion these features afford the plants to be assigned status of species and we describe it below.

### Taxonomic treatment

**Table:** 

**Figure 4:**
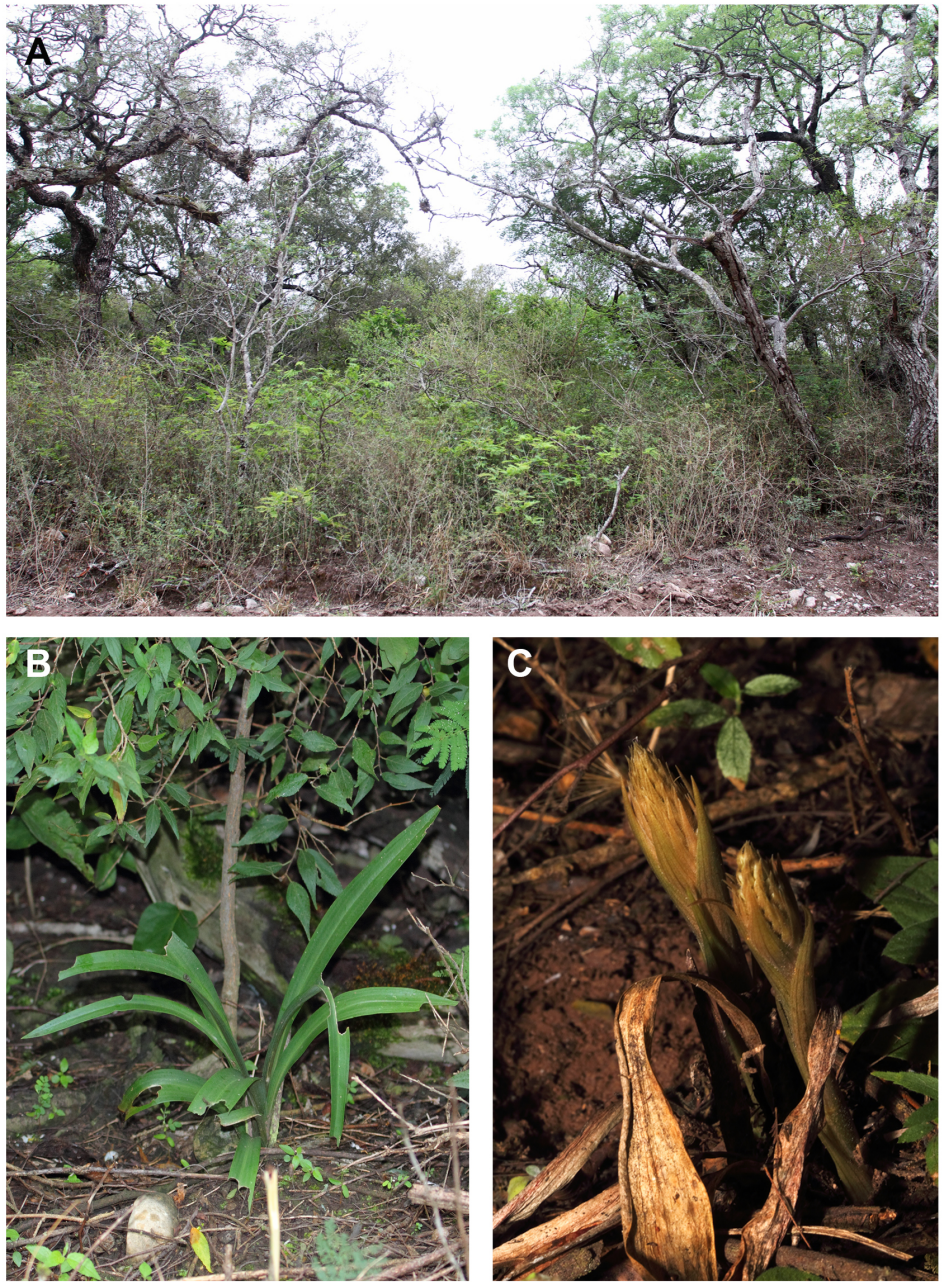
*Pachygenium laurense in situ*. (A) Habitat. (B) Leaves. (C) Inflorescence buds (photos by Claudia M. Martín).

**Figure 5:**
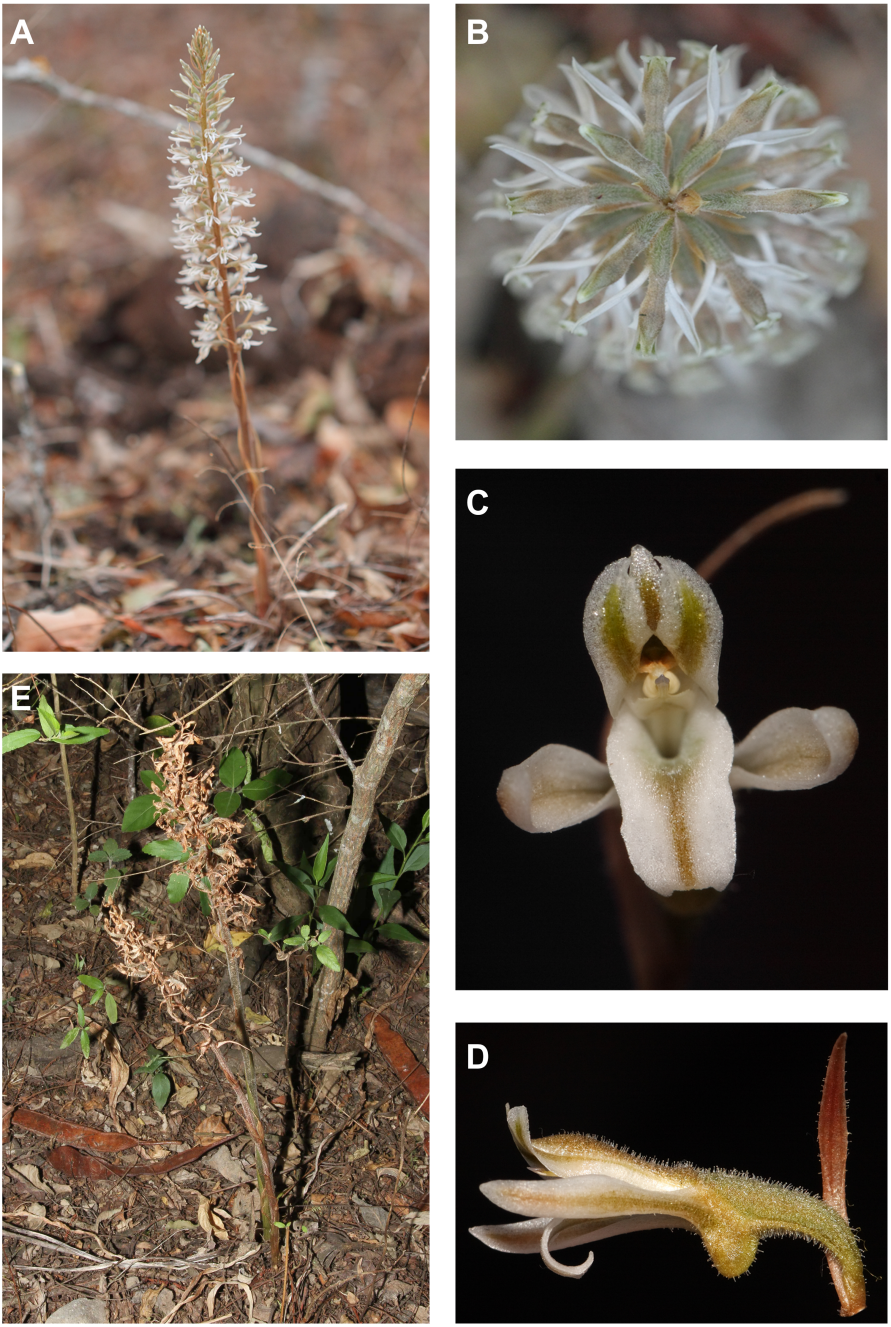
Floral structures of *Pachygenium laurense*. (A) (E) Inflorescence, lateral view (B) Inflorescence, top view. (C) Flower, front view. (D) Flower, lateral view (photos by Claudia M. Martín).

**Figure 6:**
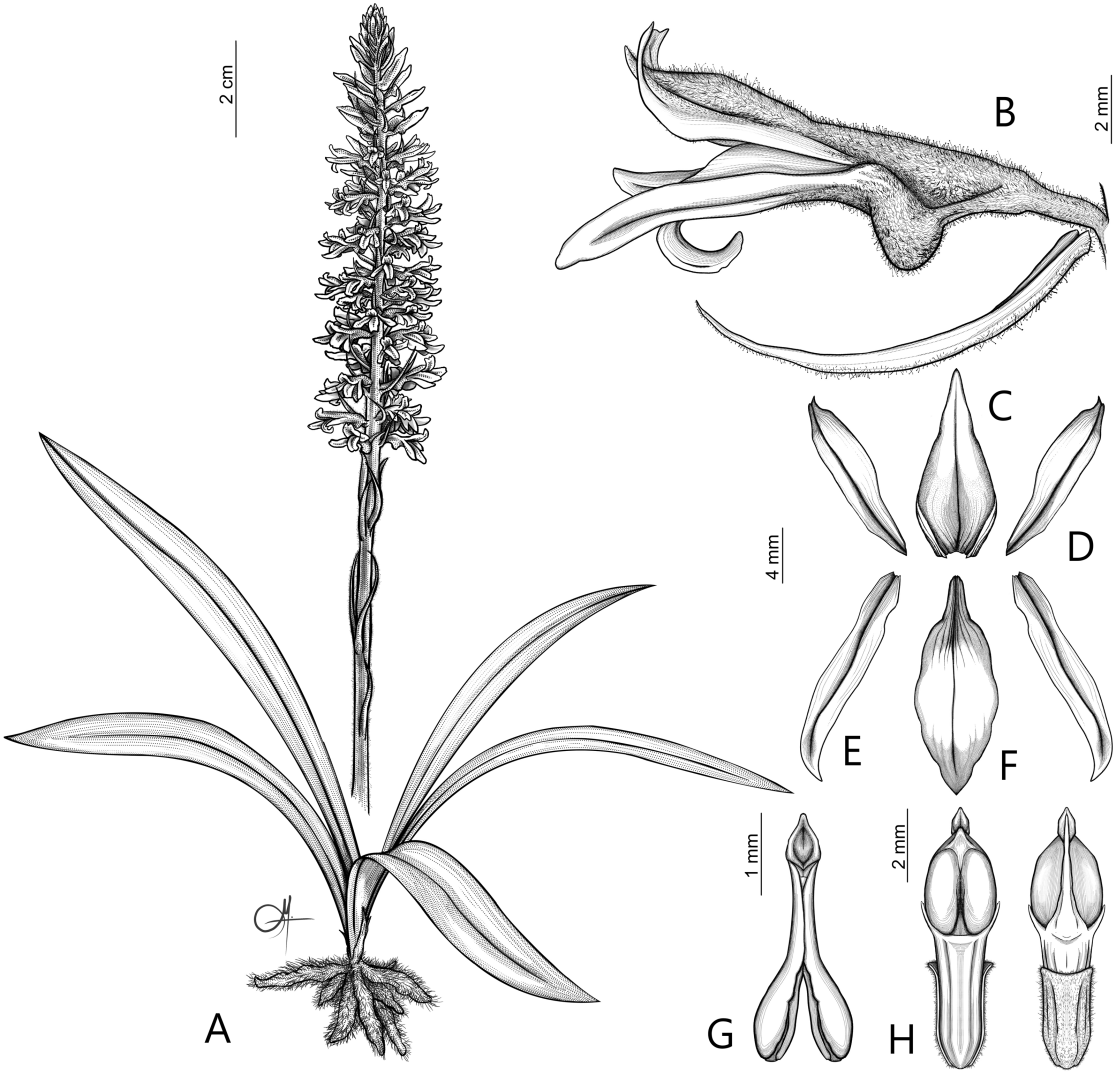
Drawing of *Pachygenium laurense*. (A) Habit. (B) Flower, lateral view. (C) Dorsal sepal. (D) Lateral sepals. (E) Petal. (F) Lip. (G) Pollinarium. (H) Gynostemium, front and back view (drawn by Adriana M. Morales).

***Pachygenium laurense*** C.M. Martín & Szlach. **sp. nov.** (Figs. 4–6)

Type: ARGENTINA. Jujuy: Dep. San Pedro, San Juan de Dios, Estancia Las Lauras, S 24°33′39″; W 64°39′26,9″, 906 msl., 03-Sep-2020, *C*.*M*. *Martín 2814* (**holotype:** JUA [JUA2814CMM]!; [UGDA-DLSz! drawing, photos]).

**Diagnosis:** Species leafless at anthesis, with very characteristic arrangements of the flowers in inflorescence, ecallose, undivided lip, and narrow, slender gynostemium.

**Figure 7:**
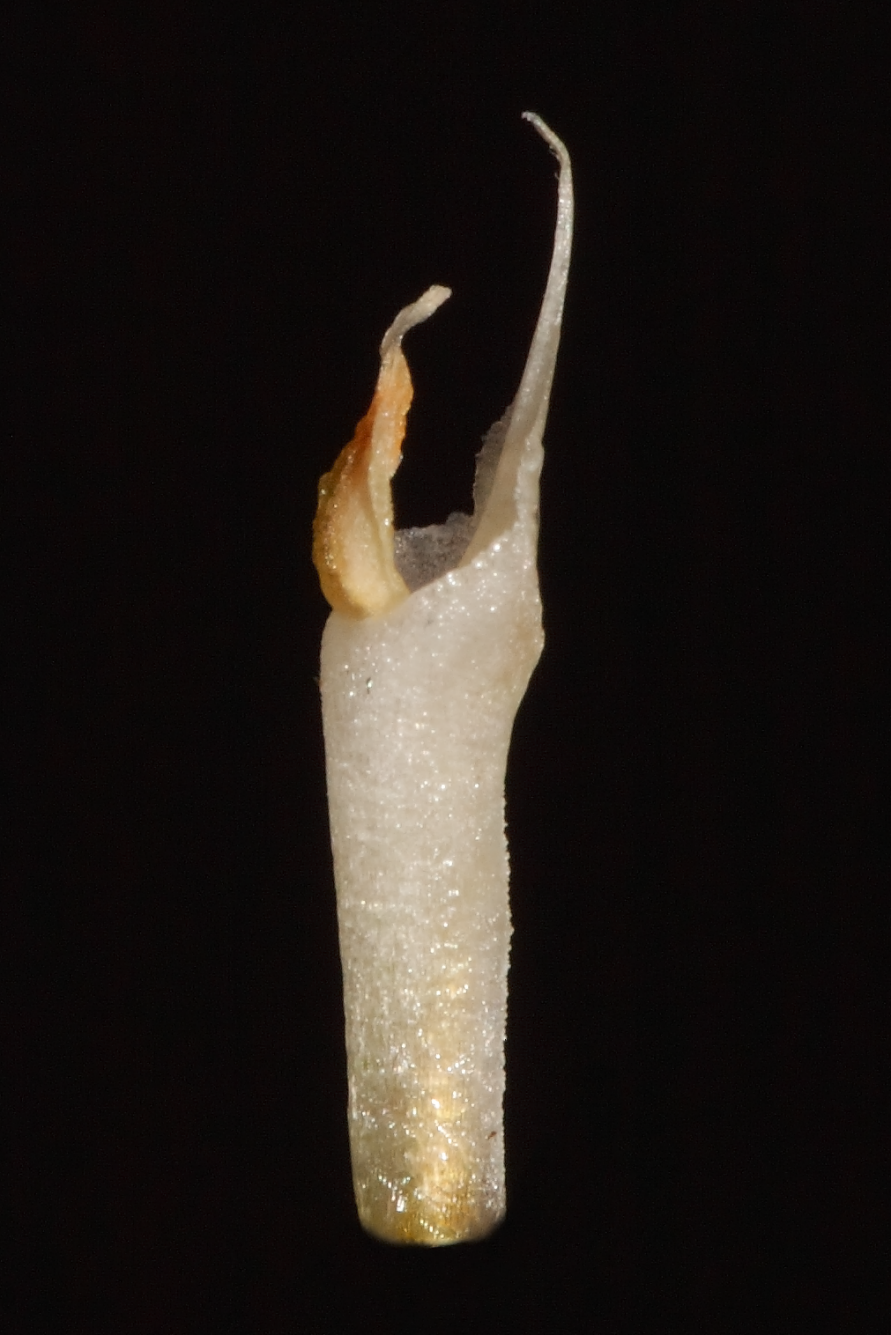
The gynostemium of *Pachygenium laurense* (photo by Claudia M. Martín).

**Description:** Plants terrestrial. Roots fasciculate, tuberous, fleshy, thin, spindle to cylindrical, hairy. Leaves 3–4(6), basal, rosulate, 22–24 × 1.5–2.8 cm, sessile, somewhat fleshy, linear-oblanceolate, acute, absent when flowering. Inflorescence erect, up 20 cm long, densely many-flowered; peduncle reddish, glandulous pubescent; sheaths 6–11, (2.5) 4 × 1 cm, the lower ones larger; rachis 16–18 cm, glandulous pubescent; floral bracts 1.8 × 0.3 cm, narrowly lanceolate, acuminate, reddish, glandulous pubescent along midvein. Flowers about 50, resupinate, rather small, cream-whitish-green in general appearance, densely glandular. Pedicellate ovary ca 0.5–0.7 cm long, densely glandular, greenish. Dorsal sepal 10 × 5 mm, ovate-lanceolate, acute, concave in the lower half, white with greenish central line on inner surface, greenish outside, densely glandular at the base, sparsely glandular above. Lateral sepals 12–14 × 2–2.5 mm, linear-lanceolate above saccate base, apically falcate, acute, white with a reddish-brown central line pubescent, decurrent on the column foot forming a chin shorter than the ovary, densely glandular at the base, sparsely above. Petals 10 × 2.2 mm, oblanceolate-ligulate above narrow base, somewhat oblique, acute, white with a greenish central line, connecting with the dorsal sepal, pubescent at the base. Lip 12 × four mm, slightly fleshy, with a narrow basal claw, elliptic-lanceolate, acute, margins somewhat wavy, ecallose, ±canaliculated in the lower half, recurved and somewhat flattened above, white with a reddish-brown central line. Gynostemium seven mm long, slender, erect, semi-fusiform, papillate on the ventral surface (Fig. 7). The anther 2 mm long, elliptic with acute apex. Pollinarium 2.5–3 mm long, basal half very narrow, slender, apical part somewhat swollen, spread. Stigma bilobed, both lobes confluent. Rostellum two mm long, terminal, linear, somewhat upcurved. Viscidium 0.5 mm long, produced on the upper surface of the rostellum, triangular-ovate in outline, acute. Clinandrium narrow.

**Etymology:** The name of the species refers to the place where it was collected for the first time (Estancia Las Lauras).

**Figure 8:**
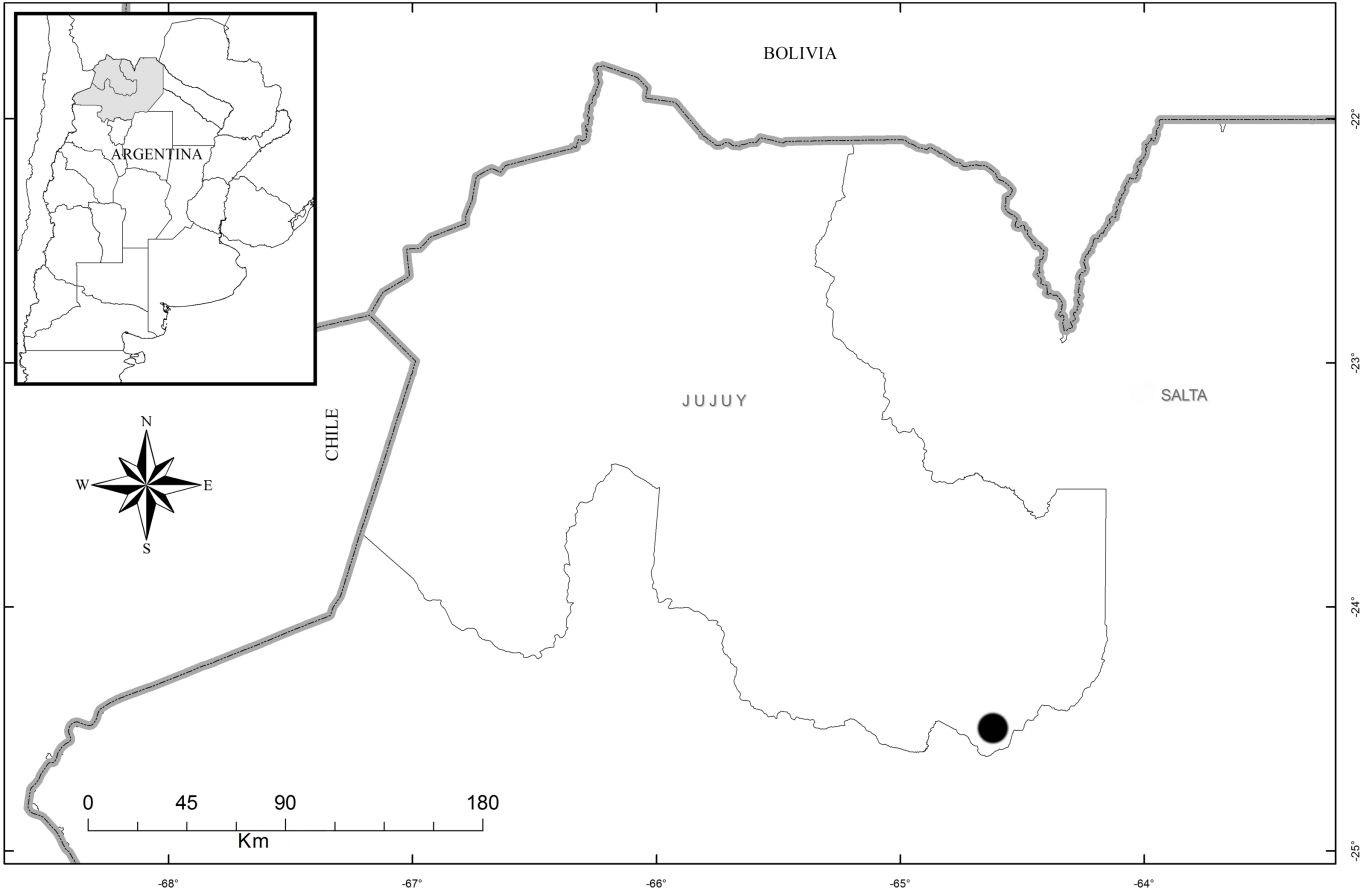
Map of the distribution of *Pachygenium laurense* (circle) (prepared by Claudia M. Martín).

**Distribution, habitat, and ecology:** The new species is restricted in distribution to the Estancia Las Lauras in San Pedro (Jujuy, Argentina) (S 24°34′16.1″; W 64°39′03.7″) (Fig. 8). The altitude of this locality is 995 m a. s. l., with a mean annual temperature from 17.7–20.2 °C, and a mean annual precipitation of 431–737 mm (Entrocassi et al., 2014). The area belongs to the phytogeographic district of the Bosque Chaqueño Occidental (Oyarzabal et al., 2018). *Pachygenium laurense* grows in the understory of a xerophilous and deciduous forest of *Schinopsis lorentzii* (Griseb.) Engl., *Alvaradoa subovata* Cronquist, *Libidibia paraguariensis* (D. Parodi) G.P. Lewis, *Gochnatia palosanto* Cabrera, *Geoffroea decorticans* (Gillies ex Hook. & Arn.) Burkart, *Ceiba chodatii* (Hassl.) Ravenna and *Athyana weinmanniifolia* (Griseb.) Radlk. Flowering occurs in the dry season, from late August to mid-September, when the plants are completely leafless. The leaves develop at the end of the rainy season.

**Information about the population:** During our studies we found less than 15 individuals, most of were growing close to each other. Thus, we considered that the individuals are aggregated and scarce.

**Conservation status:** *Pachygenium laurense* has only been recorded for the type locality, in an area smaller than 1 km^2^. In addition, the surrounding Chaco forests, where it is probable to find more populations of *P. laurense*, are being replaced by crops or livestock. For these reasons, the new species is classified as Critically Endangered CR [criteria C2a(i)] according to the World Conservation Union Red List Categories and Criteria (IUCN, 2019).

**Figure 9:**
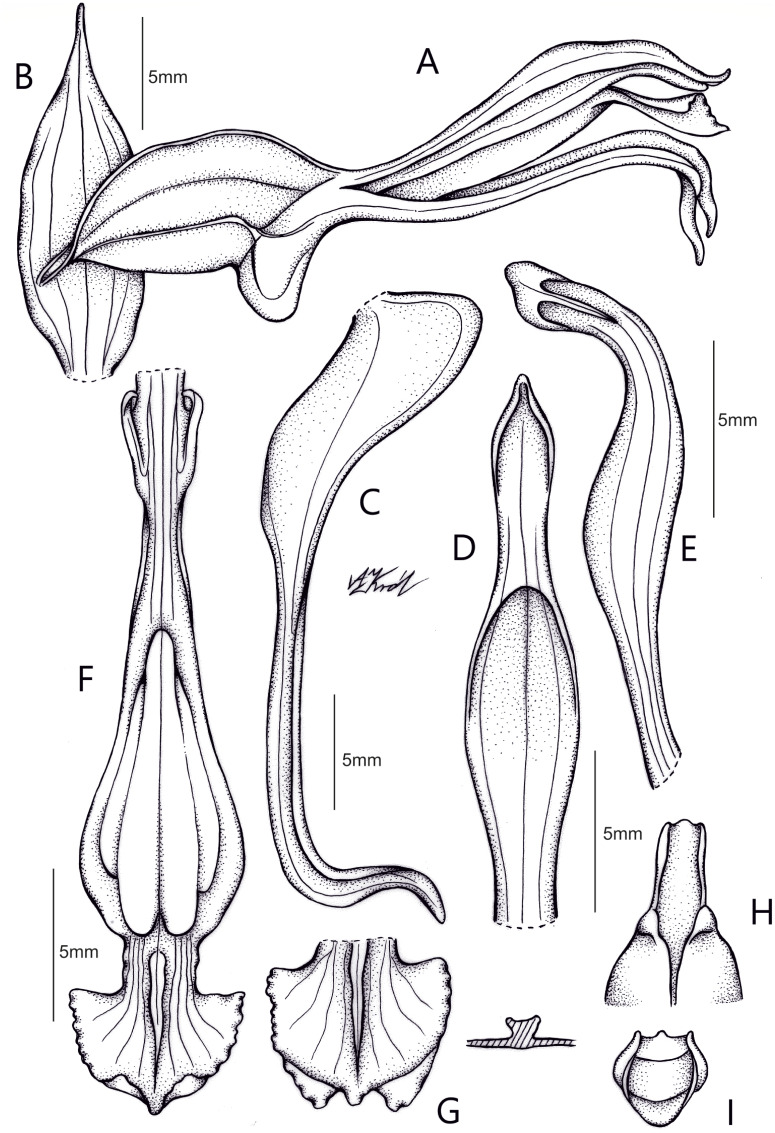
Drawing of *Pachygenium gutturosa* com. nov. from type material *H*. *Wendland 427* (W-R35230). (A) Flower, side view. (B) Floral bract. (C) Lateral sepal. (D) Dorsal sepal. (E) Petal. (F) Lip. (G) Apical part of lip. (H) Gynostemium apex, bottom view. (I) Viscidium (drawn by Anna Król).

***Pachygenium gutturosa*** (Rchb.f.) Szlach., Dudek & C.M. Martín, **comb. nov.** (Fig. 9)

Basionym: *Spiranthes gutturosa* Rchb.f., Beitr. Orchid.-K. C. Amer. 67. 1866; Type: EL SALVADOR, St. Vincent, 13 Feb 1857, *H*. *Wendland 427* (holotype: W-R!). ≡ *Sarcoglottis gutturosa* (Rchb.f.) Ames ex Donn. Sm., Enum. Pl. Guatem. 7: 49. 1905. ≡ *Pelexia gutturosa* (Rchb.f.) Garay, Bot. Mus. Leafl. 28(4): 344. 1980-1982.

### Key to the species of *Pachygenium* in Argentina

**Table:** 

1. Plant leafless at anthesis, lip entire, undivided, elliptic-lanceolate, acute, without basal auricles ……………………………………………………………………………... *P. laurense*
1* Plant leafy at anthesis, lip ±constricted, basally auriculated, lamina not above …………………………………………………………………………………………2
2. Leaves oblong-obovate to elliptic-obovate, 5–6 cm wide ……………………... *P. ovatifolium*
2* Leaves not as above …………………………………………………………………... 3
3. Lip lamina narrow, strip-like in a basal part, much expanded above, ±ovate ………………………………………………………………………………*P. paludosum*
3* Lip not as above ……………………………………………………………………….... 4
4. Petals constricted in the apical 5th or 6th, apex obliquely rhombic, acute ……….. *P. saltense*
4* Petals oblong ligulate, obtuse ……………………………………………………………5
5. Lip distinctly constricted, hypochile oblong triangular to ovate ………………. *P. bonariense*
5* Lip somewhat constricted, hypochile oblong ……………………………………. *P. ekmanii*

##  Supplemental Information

10.7717/peerj.13433/supp-1 Supplemental Information 1 A list of the taxa with their accession numbers were used on phylogenetic analyses

## Additional Information and Declarations

### Field Study Permissions

The following information was supplied relating to field study approvals (i.e., approving body and any reference numbers): The research presented here adhered to all provincial and national laws. The Mustad family gave us permission to investigate at Estancia Las Lauras. Also, the Dirección Provincial de Biodiversidad de Jujuy–Secretaría de Gestión Ambiental (Jujuy, Argentina) provided permission to conduct the research under permit number 171/2015-DPB.

### Data Availability

The following information was supplied regarding data availability: The ITS, matK and trnL-trnF sequences described here are available at GenBank: OL619327 to OL619330; OL694861 to OL694863, and OL631599 to OL631602.

### New Species Registration

The following information was supplied regarding the registration of a newly described species: Pachygenium laurense C.M. Martín & Szlach. sp. nov.: 77297261-1 Pachygenium gutturosa (Rchb.f.) Szlach., Dudek & C.M. Martín: 77297263-1
