# Supplementary material for: Pachygenium laurense (Orchidaceae, Spiranthinae), a new orchid species from Argentina—morphological evidence and phylogenetic reconstruction
Source: PeerJ. 2022 May 26;10:e13433. doi: 10.7717/peerj.13433 (PMC9148561; doi:10.7717/peerj.13433)
Supplement: Supplemental Information 1 [file peerj-10-13433-s001.docx]

**A list of the taxa with their accession numbers were used on phylogenetic analyses.**

| **taxon** | **accesion number of ITS** | **accesion numer of matK** | **accesion numer of trnL-trnF** |
| --- | --- | --- | --- |
| Cyclopogon apricus | KX421924.1 | KX421978.1 | KX422031.1 |
| Cyclopogon calophyllus | KX421930.1 | KX421983.1 | KX422037.1 |
| Cyclopogon carinianus | KX421931.1 | ------ | ------ |
| Cyclopogon congestus | KX421925.1 | KX421980.1 | KX422033.1 |
| Cyclopogon elegans | KX421934.1 | ------ | KX422040.1 |
| Cyclopogon eugenii | KX421935.1 | ------ | KX422041.1 |
| Cyclopogon obliquus | MF465000.1 | MG755116.1 | MG582357.1 |
| Cyclopogon variegatus | KX421936.1 | ------ | KX422042.1 |
| Cyclopogon warmingii | KX421938.1 | KX421987.1 | KX422043.1 |
| Brachystele bracteosa | KX421957.1 | KX422008.1 | ------ |
| Brachystele camporum1 | KX421960.1 | KX422009.1 | KX422067.1 |
| Brachystele camporum2 | KX421958.1 | ------ | ------ |
| Brachystele dilatata1 | KX421962.1 | KX422012.1 | KX422069.1 |
| Brachystele dilatata2 | KX421961.1 | ------ | KX422068.1 |
| Brachystele subfiliformis | KX421964.1 | KX422015.1 | KX422071.1 |
| Brachystele ulaei | KX421965.1 | KX422016.1 | ------ |
| Brachystele unilateralis1 | KX421967.1 | KX422017.1 | KX422074.1 |
| Brachystele unilateralis2 | KX421966.1 | ------ | KX422073.1 |
| Brachystele widgrenii | KX421968.1 | KX422019.1 | KX422075.1 |
| Glohisarcon weberbaueri1 | MF464952.1 | MG755146.1 | MG582311.1 |
| Glohisarcon weberbaueri2 | EF079169.1 | EU395448.1 | ------ |
| Pachygenium bonariensis | MF464958.1 | MG755132.1 | MG582316.1 |
| Pachygenium cf tamanduensis | KX421944.1 | ------ | ------ |
| Pachygenium ekmanii1 | KX421945.1 | KX421994.1 | KX422051.1 |
| Pachygenium ekmanii2 | MF464959.1 | MG755134.1 | MG582317.1 |
| Pachygenium gutturosa | MF464962.1 | MG755137.1 | MG582319.1 |
| Pachygenium hirta | MF464960.1 | MG755138.1 | ------ |
| Pachygenium laminata1 | KX421948.1 | KX421997.1 | KX422054.1 |
| Pachygenium laminata2 | KX421947.1 | KX421996.1 | KX422053.1 |
| Pachygenuim minarum | MG738360.1 | MG755143.1 | MG734369.1 |
| Pachygenium oestrifera | KX421949.1 | KX421998.1 | KX422055.1 |
| Pachygenium orobanchoides1 | MG460388.1 | MG460453.1 | MG460420.1 |
| Pachygenium orobanchoides2 | MG460387.1 | MG460452.1 | MG460419.1 |
| Pachygenium orthosepala1 | KX421951.1 | KX422000.1 | KX422056.1 |
| Pachygenium orthosepala2 | KX421950.1 | MG755145.1 | MG582318.1 |
| Pachygenium orthosepala3 | MF464961.1 | ------ | ------ |
| Pachygenium parva | KX421952.1 | KX422002.1 | KX422059.1 |
| Pachygenium paludosa | ------ | KX422001.1 | KX422058.1 |
| Pachygenium pterygantha | KX421943.1 | KX421992.1 | KX422049.1 |
| Pachygenium tamanduensis | ------ | ------ | KX422050.1 |
| Pachgenium ventricosa | MG460389.1 | MG460454.1 | MG460421.1 |
| Pelexia adnata | AJ539501.1 | ------ | ------ |
| Pelexia congesta | MF464966.1 | MG755133.1 | MG582323.1 |
| Pelexia funckiana | MF464965.1 | MG755135.1 | MG582322.1 |
| Pelexia goninensis | MF464964.1 | MG755136.1 | MG582321.1 |
| Pelexia hondurensis1 | MF464968.1 | MG755140.1 | MG582325.1 |
| Pelexia hondurensis2 | MF464967.1 | MG755139.1 | MG582324.1 |
| Pelexia lindmanii | MF464963.1 | MG755141.1 | MG582320.1 |
| Pelexia maculata1 | FJ473335.1 | ------ | FJ571287.1 |
| Pelexia maculata2 | MF464969.1 | MG755142.1 | MG582326.1 |
| Pelexia macropoda1 | KX421955.1 | KX422005.1 | KX422063.1 |
| Pelexia macropoda2 | KX421954.1 | KX422006.1 | KX422062.1 |
| Pelexia novofriburgensis | MG460386.1 | MG460451.1 | MG460418.1 |
| Pelexia novofriburgensis | KX421953.1 | KX422004.1 | KX422061.1 |
| Pelexia olivacea | MF464970.1 | MG755144.1 | MG582327.1 |
| Pelexia sp. | ------ | ------ | MG582329.1 |
| Sarcoglottis acaulis1 | KJ472391.1 | KJ472359.1 | ------ |
| Sarcoglottis acaulis2 | FJ473349.1 | EU395447.1 | FJ571300.1 |
| Sarcoglottis assurgens | MF464986.1 | MG755152.1 | MG582343.1 |
| Sarcoglottis biflora | MG460394.1 | MG518229.1 | MG460427.1 |
| Sarcoglottis cerina | MF464980.1 | MG755153.1 | MG582337.1 |
| Sarcoglottis corymbosa | MF464985.1 | MG755154.1 | MG582342.1 |
| Sarcoglottis curvisepala | MF464974.1 | MG755155.1 | MG582331.1 |
| Sarcoglottis fasciculata | KX421919.1 | MG755156.1 | MG582335.1 |
| Sarcoglottis grandiflora | FJ473350.1 | ------ | FJ571301.1 |
| Sarcoglottis grandiflora | MF464973.1 | MG755157.1 | MG582330.1 |
| Sarcoglottis homalogastra | MG460395.1 | MG460461.1 | MG460428.1 |
| Sarcoglottis lobata | MF464981.1 | MG755158.1 | MG582338.1 |
| Sarcoglottis neglecta | ------ | EF079292.1 | ------ |
| Sarcoglottis portillae | EF079167.1 | ------ | ------ |
| Sarcoglottis sancta | ------ | KX421972.1 | KX422024.1 |
| Sarcoglottis sceptrodes | MF464975.1 | MG755162.1 | MG582332.1 |
| Sarcoglottis scintillans | MF464984.1 | MG755164.1 | MG582341.1 |
| Sarcoglottis schwackei | KX421918.1 | KX421975.1 | MG460429.1 |
| Sarcoglottis schaffneri | AM778171.1 | AM902106.1 | MG582345.1 |
| Sarcoglottis smithii | MF464982.1 | MG755165.1 | MG582339.1 |
| Sarcoglottis speciosa | MF464976.1 | MG755166.1 | MG582333.1 |
| Sarcoglottis rosulata | MF464987.1 | MG755161.1 | MG582344.1 |
| Sarcoglottis richardiana | MF464983.1 | MG755159.1 |  |
| Sarcoglottis riocontensis | KX421920.1 | KX421976.1 | KX422028.1 |
| Sarcoglottis ventricosa | MF464972.1 | ------ | ------ |
| Sarcoglottis uliginosa | KX421921.1 | KX421977.1 | KX422029.1 |
| Veyretia hassleri | KX421940.1 | KX421988.1 | KX422045.1 |
| Veyretia simplex | KX421942.1 | KX421991.1 | KX422048.1 |
| Veyretia sincorensis | KX421941.1 | KX421990.1 | KX422047.1 |
